# Anti-Aging Effects of Flavonoids from Plant Extracts

**DOI:** 10.3390/foods13152441

**Published:** 2024-08-02

**Authors:** Bogdan Păcularu-Burada, Alexandru-Ionuț Cîrîc, Mihaela Begea

**Affiliations:** 1 ICA Research & Development S.R.L., 202 Splaiul Independenței, 060021 Bucharest, Romania; bogdan.pacularu@ica-rd.ro; 2 Dan Voiculescu Foundation for the Development of Romania, 011885 Bucharest, Romania; 3 Faculty of Biotechnical Systems Engineering, National University of Science and Technology Politehnica Bucharest, 313 Splaiul Independenței, 060042 Bucharest, Romania; mihaela.begea@upb.ro

**Keywords:** flavonoids, senescence, diseases, anti-aging, nutraceuticals

## Abstract

Aging is a natural and irreversible process, affecting living organisms by negatively impacting the tissues’ and cells’ morphology and functionality and consequently being responsible for aging-related diseases. Taking into account the actual preoccupations of both consumers and researchers, healthy anti-aging alternatives are being intensively studied in order to address such concerns. Due to their functional features, plant flavonoids can be considered valuable nutraceuticals. This paper highlights the possibilities to use flavonoids extracted from various plants for their anti-aging potential on the skin, brain, and heart. Moreover, their anticarcinogenic, anti-inflammatory, and anti-diabetic properties are summarized, along with the senescence-associated mechanisms. Both the nutraceutical and cosmeceutical fields are continuously developing and flavonoids originating from plants are promising candidates to obtain such products. Thus, the bioactive compounds’ extraction and their subsequent involvement in innovative product manufacturing must be carefully performed while being aware of the various intrinsic and extrinsic factors that may affect the phytochemicals’ structures, bioavailability, and health effects.

## 1. Introduction

Aging is a natural process occurring in living organisms, characterized by the modification of normal cells’ functions and morphologies. As a consequence, senescent cells’ excessive accumulation in tissue is a risk factor for aging-related diseases [1,2]. Some metabolic diseases that are simultaneously associated with aging can be easily identified and managed by assessing specific biomarkers (e.g., pro-inflammatory cytokines—tumor necrosis factor-alpha (TNF-α) and interleukin-6 (IL-6)). These cytokines have been determined in patients with diabetes, cardiovascular disease, and fatty liver [3]. Moreover, the inflammatory reactions that are correlated with diseases can be appreciated via specific interleukins’ levels or enzymes’ activity. Specifically, IL-6, IL-8, IL-1β, IL-17, cyclooxygenase-2 (COX-2), lipoxygenases, and nitric oxide synthase are responsible for inflammation [4]. 

Some pharmaceuticals (e.g., metformin, rapamycin, nicotinamide mononucleotide, and α-ketoglutarate) have been tested *in vivo* on animal models or included in clinical trials to demonstrate their anti-aging effects [5]. Their outcomes depend on multiple variables and their utilization may be the cause of some health-related side effects [6]. In this sense, the actual concerns of consumers and researchers revolve around identifying natural and sustainable sources of biologically active compounds that could be included further in various products to maximize their positive effects [7].

Nutraceuticals are defined as foods, food components, or dietary supplements with medical and health benefits, including the prevention and treatment of diseases [8,9]. However, nutraceuticals’ definition is still under debate in terms of establishing an international consensus. Some authors suggest that nutraceuticals can be classified into traditional and non-traditional ones. Chemical constituents (nutrients, herbals, fatty acids, phytochemicals), prebiotics, probiotics, and enzymes are included in traditional nutraceuticals, whereas fortified and recombinant nutraceuticals are included in non-traditional nutraceuticals [10]. Nutraceuticals are extracted from foods, nutrients, or herbs. They can exert various therapeutic properties (i.e., antioxidant, anti-inflammatory, immunomodulatory, and anticarcinogenic) [11]. The main advantages of nutraceuticals’ administration rely on their targeted action towards specific disorders, as well as an improved effect due to the concentrated form of the compound(s) within ready-to-use products. Interestingly, the beneficial effects can be maximized by designing multi-component formulas using different raw materials [12] and consequently obtaining non-traditional nutraceuticals. Nutraceuticals must be administered as pills, capsules, syrups, gels, or extracts for a specific period of time in order to exert positive health benefits. The regulatory aspects of nutraceuticals depend on the geographical area. In particular, in the European Union, nutraceuticals are included in dietary supplements, which are regulated by Directive 2002/46/EC under the supervision of the European Food Safety Authority (EFSA) [13]. The further uses of nutraceutical formulas in foods, including flavonoid-rich nutraceuticals, must follow EC 1333/2008 [14] regarding authorized food additives with antioxidant effects. Therefore, EFSA is continuously updating the lists of food additives and some differences may occur among countries based on the national legislation [15]. In contrast, the Dietary Supplement, Health and Education Act of 1994 is partly responsible for dietary supplements’ and nutraceuticals’ use in the United States. The Food and Drug Administration (FDA) requires in-depth preclinical and clinical trials to demonstrate the safety aspects of these compounds and increase their marketability [16]. 

Flavonoid compounds are types of polyphenols, which are frequently found in plants, fruits, vegetables, cereals, and legumes. The interest regarding these biomolecules lies in their functional properties, which make them suitable for nutraceutical formulations [17,18]. Specific flavonoids, such as quercetin, kaempferol, curcumin, myricetin, anthocyanins, or catechins, are beneficial in Alzheimer’s disease, diabetes, or hypertension and cardiovascular disease [3,19,20]. Furthermore, quercetin, hesperidin, and delphinidin were included in flavonoid-based compositions for skin aging prevention and treatment. For these cosmetic products, the phytochemicals’ stability was improved through micro/nanocapsules or cream preparations [21]. The anti-cancer effects of some plant flavonoids were summarized by Rahaman et al. [22], highlighting that quercetin, luteolin, and kaempferol, along with other flavonoids, possess these properties due to their COX-2 and matrix metalloprotease (MMP) inhibition potential; thus, the inhibitory mechanism differs among compounds and cancer types.

This review paper aims at briefly summarizing the potential uses of unexploited plant species for their richness in flavonoids. Furthermore, the applications of flavonoid-based formulations, as nutraceuticals with anti-aging effects, are highlighted. The positive impacts of flavonoids and their mechanisms of action are discussed considering the latest published articles in the field. 

## 2. Aging Mechanisms and Flavonoids

Aging is characterized by the presence of senescent cells, and their impact is well documented nowadays [23]. Such scientific studies are helpful because specific biomarkers (p16^INK4A^, p21^CIP1^, p27, p19^ARF^, p53, and PAI-1) allow the identification of modified cells in tissues and organs [24]. Moreover, the senescence process is correlated with the modification of the senescence-associated secretory phenotype (SASP). The SASP consists of specialized components associated with the aging process, like pro-inflammatory interleukins (IL-6, IL-8, and IL-1α), chemokines (CXCL-2, CXCL-3, CXCL-5), or MMP-1 and MMP-3 [25,26]. The senescent cells play a crucial role in signaling and the healing of aging-associated conditions affecting the skin, bones, lungs, liver, brain, heart, or eyes after prolonged exposure to oxidative stress [27]. Furthermore, the symptoms of rheumatoid arthritis, mainly chronic joints’ inflammation, could be alleviated after flavonoid administration *in vitro* or *in vivo*. Quercetin, epigallocatechin-3-gallate, naringenin, and naringin positively changed the cytokine levels produced by T cells (e.g., Th 1, Th 2, and Th 17), demonstrating both anti-inflammatory and anti-aging properties [28]. Collagen production was improved due to the luteolin-inhibitory capacity on MMPs, which supports tissue regeneration. The same beneficial effect on joints’ health was demonstrated by kaempferol when doses of 100 µM, ranging between 20 and 200 mg/kg, were tested *in vivo* on rats, concluding that such outcomes are possible because of the binding capacity of kaempferol with free radicals and proteins [29]. *In vivo* studies on rats suggest that articular injections with apigenin (50 µL, once a week for 3 weeks) or quercetin (100 µL, once a week for 6 weeks) contribute to pro-inflammatory cytokines’ reduction and articular tissue regeneration [30]. The anti-aging benefits of flavonoids on joints are related to these compounds’ ability to reduce MMPs, IL-1β, COX-2, and TNF-α [31]. Interestingly, TNF-α was identified as a biomarker for muscle health and aging. In this sense, a reduction in TNF-α was observed in mice with muscle atrophy after quercetin administration. Luteolin, epigallocatechin gallate, and epicatechin administration in mice prevents muscle degradation by supporting normal mitochondrial DNA activity, muscle mass improvement, and repair, respectively [32]. Tea catechin (540 mg/day, twice a week for 3 months) or epicatechin capsules (1 mg/kg body weight (BW)/day for 8 weeks) improved muscular mass and strength in the clinical studies summarized by Wu and Suzuki [33]. *In vivo* studies on mice confirm the above-mentioned capacities of catechins and their derivatives, along with quercetin, hesperidin, and apigenin, which act on mitochondria and ensure their normal functioning and satisfy the oxygen requirements, respectively, reducing the oxidative reactions on muscle fibrils, thus preventing inflammation and aging [34,35]. 

Reactive radicals or non-radical derivatives of oxygen and nitrogen are known as reactive oxygen species (ROS) and reactive nitrogen species (RNS), both comprising the reactive oxygen and nitrogen species (RONS) directly involved in energy regulation, signaling pathways, immunity, aging, and aging-related diseases. RONS appear as a result of specific enzymes, such as oxidases, peroxidases, and angiotensin II, as the main endogenous sources. Environmental factors and pollution, along with an unhealthy lifestyle, are the main causes of exogenous RONS [36]. During mitochondrial metabolism, oxygen is consumed and ROS are generated. Modifications of this process result in the activation of oxidative stress mechanisms, which are precursors of aging-associated conditions, because of the cellular components’ mutations, such as DNA, proteins, or fatty acids [37]. 

**Figure 1:**
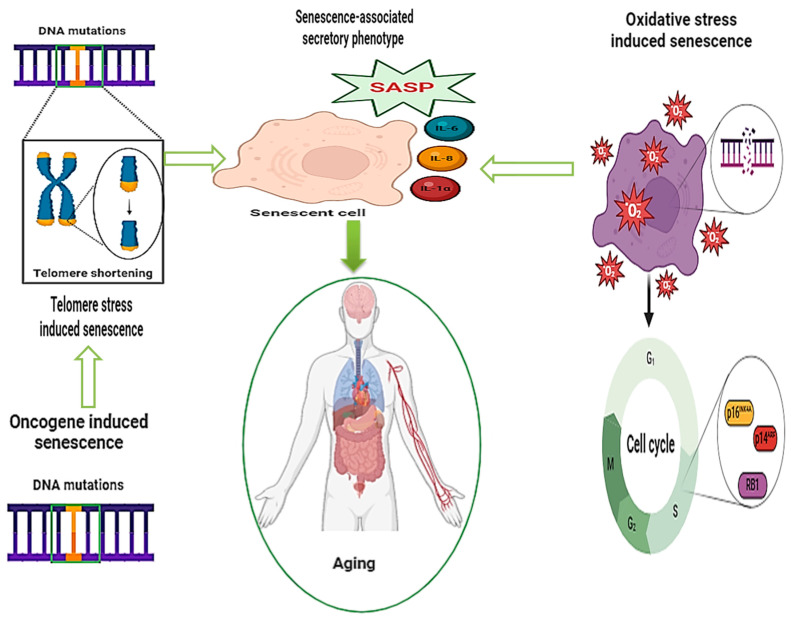
Senescence mechanisms attributed to aging-related conditions (adapted from Fabregat et al. [39]).

The SASP is modified by RONS, which determine (i) a pro-inflammatory reaction and tumor proliferation due to the increased production of specific interleukins (IL-1α); (ii) an inhibitory effect on superoxide dismutase (SOD), affecting TNF-α receptors; (iii) the decreased protection of insulin/insulin-like growth factors (IGF) against oxidative stress; (iv) increased levels of MMPs associated with chronic and age-related conditions; and (v) alterations of specific pathways involving p16^INK4a^, p53, and p21 as a result of senescence [38], as shown in Figure 1. 

Senescence also modifies the immune response. Innate immunity is associated with cytokine and chemokine production, known as specific receptors for inflammation (IL-1, IL-18, IL-33), or interferon production, whereas T-cells regulate adaptive immunity. The information summarized by Fulop et al. [40] suggests that specific reactions occur as a result of inflammation determined by aging, also known as inflammaging [41]. On one hand, modifications of the free radicals were observed, as well as an increment in cytokines’ and myeloids’ numbers, attributed to the innate immunity. On the other hand, a decrease in the naïve T-cells and immunoglobulin production was determined, along with an increase in memory T-cells, all of them being part of the adaptive immune system. Such alterations were attributed to age-related inflammatory reactions as an effect of mitochondrial dysfunction, DNA damage, or SASP system modifications of the senescent T-cells. Fortunately, some flavonoids (e.g., hesperidin, apigenin, catechin, kaempferol, and anthocyanins) intervene in the adaptive and innate immunity and prevent inflammatory reactions [42]. Aging multi-omics is a novel approach that is able to predict and identify specific aging-associated metabolic reactions. Hence, proteomics was successfully used to identify and predict aging and its related disorders. The expression of aging-related interleukins, such as IL-6 and IL-8, as well as protein kinases within the AMP-activated protein kinase (AMPK) pathway and reductases’ or telomerases’ activity, was studied in order to clarify their implications in the aging process, emphasizing that some play an essential role as aging biomarkers, while others are associated with cellular senescence and life cycle, lifespan, or oxidative stress [43].

Cellular longevity is attributed to proper autophagy, a process in which catabolic metabolites, especially degraded proteins and other organelles, are excreted from cells to prevent toxic compounds’ accumulation. Alterations of this process due to various kinases’ alterations may lead to exaggerated cell proliferation or cellular death, which can lead to dysfunction of the tissue and organs, leading to age-related conditions [44]. In addition, apoptosis is a normal cellular process employing caspases (caspase-3, caspase-6, caspase-7, and caspase-8), which are able to maintain cellular homeostasis by regulating mitochondrial and DNA-related processes. Therefore, stress can affect the caspases’ activity. As a consequence, the cellular senescence and aging processes are accelerated [45].

Skin aging occurs due to ROS accumulation in the dermal cells. As a consequence, the mitogen-activated protein kinase (MAPK) activity within the fibroblasts is negatively affected. This alteration, on one hand, decreases the production of collagen. On the other hand, collagen degradation and its fragmentation occur as a consequence of the overexpression of MMP-1, MMP-3, and MMP-9. Moreover, it was demonstrated that the elastic fibers of the skin are affected by UV light, which increases the risk of wrinkles, such outcomes being associated with the overexpression of MMP-12 [46]. Besides fibroblasts, the skin comprises keratinocytes, which change their size and shape with aging, loosing their elasticity and vascularization. A specific interleukin, namely IL-1α, was established as a biomarker for senescent keratinocytes. Additionally, melanocytes, responsible for skin pigmentation, which is accentuated with aging, produce high levels of melanin as an effect of high tyrosinase activity, correlated with the decreased synthesis of melanocytes. Such aging-associated effects of these cells could be justified by the weak melanocyte protection mechanisms upon UV light exposure over time [47].

Brain aging affects memory, learning, and cognitive and intellectual function, which is the main side effect of proteostasis or neuronal autophagy abnormalities and dysfunctionalities. The alterations of brain function associated with aging rely also on decreased synaptic plasticity and oxidative stress, which promote inflammatory reactions regulated by specific pro-inflammatory cytokines such as IL-6, IL-1β, or TNF-α [48]. 

Aging modifies the circulatory system, involving structural and functional alterations, which can be frequently observed in the cerebral vasculature. The inflammatory reactions in the brain may be the cause of microcirculatory lesions, other important types of dysfunction being related to microvascular injuries and modified cortical function. All of these previously mentioned factors, along with diabetes, hypertension, obesity, and arterial-associated conditions, increase the chances of stroke [49]. The experimental study conducted by Sayed et al. [50] highlighted that specific proteins are responsible for the cardiovascular system’s aging. Specifically, the chemokine CXCL9 seemed to be directly involved in the cardiac aging process by affecting cardiac remodeling and cardiovascular function. Cardiovascular aging mechanisms were documented by Ungvari et al. [51], concluding that oxidative stress leads to pro-inflammatory reactions by changing the levels of MMPs, which negatively impacts the arterial structure and morphology. Additionally, the mitochondrial ROS contribute to age-related vascular dysfunction by inhibiting target SODs or via mitochondrial DNA alterations. Interleukins (IL-6, IL-1β, and TNF-α) are directly involved in vascular-associated conditions by promoting cellular apoptosis due to the negative changes in metabolic function. Flavonoids can inhibit COXs or angiotensin-converting enzyme (ACE). As such, a blood pressure and platelet function reduction has been reported [52]. 

Cancers may occur as a result of cellular aging and death; as a consequence of p^16INK4A^ tumor suppressor genes, which determine cell arrest; or due to the p53 effector protein, able to activate pro-apoptotic genes, causing cell death. As a result, the ROS levels increase and the MAPK pathway is negatively modified [53]. The excessive activity of the P13K/AKT pathway determines the overexpression of the p53 protein, which seems to be directly associated with oncogene-induced senescence [54]. Senescence is a natural and normal process occurring in living cells. In healthy organisms, senescent cells have a positive role by acting on pre-malignant cells and by suppressing tumors. Therefore, these senescent cells liberate cancer biomarkers, such as the p16^INK4A^ and p21 proteins or β-galactose, which were successfully identified in breast and colon cancer. Their identification was possible after pre-malignant cells’ mutation, along with abnormal proliferation [55,56]. It is important to mention that the abnormalities in the SASP-induced senescence mechanisms can be characterized by aging induction in the normal cells around the senescent ones [57]. Excessive mitochondrial activity due to high levels of ROS directly affects telomerase function and determines telomeres’ shortening. The same negative effect may be observed as a result of the SASP [58]. Overall, short telomeres are risk factors for age-related conditions affecting the skin, lungs, brain, heart, pancreas, bones, or immune system [59,60]. Some specific flavonoids, such as genistein or catechins, are able to exert inhibitory activity on telomerases and prevent telomere shortening, with anti-aging potential for the skin thanks to their antioxidant features [61].

**Figure 2:**
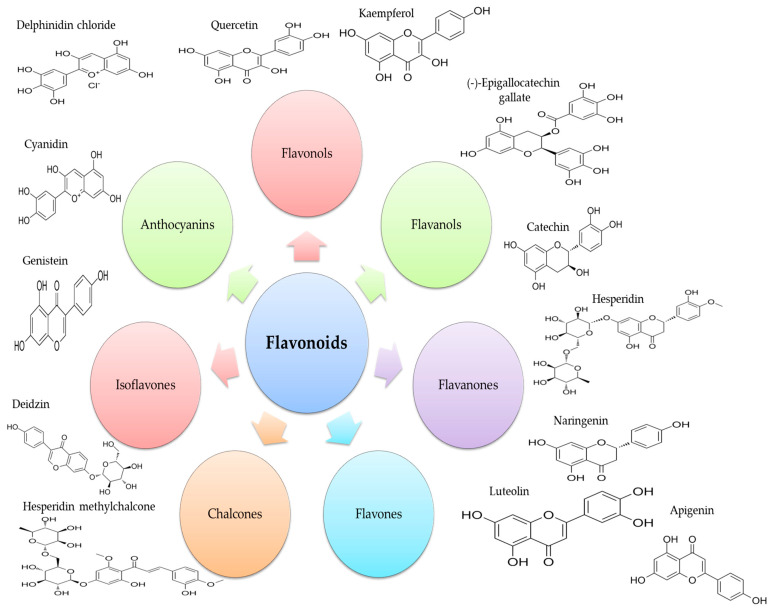
Flavonoid classes and main representatives (adapted from Chen et al. [67]).

Common dietary sources of flavonoids are green tea, red wine, fruits, and vegetables. Moreover, flavonoids are responsible for the colors of some plants and flowers, playing an important role in their protection mechanisms against environmental stress conditions [62]. Studies suggest that flavonoids’ synthesis in plants needs acetic acid and phenylalanine, which are further involved in the phenylpropanoid pathway [52]. During these metabolic reactions, hydroxylases, isomerases, reductases, and metal ions exert their activity to form various classes of compounds [63]. Considering as the main classification criterion their structure—specifically the nature of the functional groups on the three aromatic rings (C6–C3–C6) and their conformations—flavonoids are divided into seven classes as follows: flavonols, flavanols, flavanones, flavones, chalcones, isoflavones, and anthocyanins (Figure 2). 

It is important to mention that the health-related benefits of flavonoids are correlated with their chemical structures. Consequently, the structure–function relationship is a decisive factor that may influence the *in vitro* or *in vivo* results. Scientific studies suggest that the double bonds between C2 and C3 (C2=C3), as well as the hydroxyl groups within the flavonoids’ structures (e.g., quercetin, kaempferol, apigenin, and luteolin), are responsible for the anti-tumor effects in cancers by maximizing the anti-proliferative properties. Additionally, glycosylation, methylation, and hydroxylation improve the protein–flavonoid interactions, boosting the nitric oxide synthase activity with cardio-protective benefits [64]. The C2=C3 bond, along with the hydroxyl groups in specific positions on the flavonoids’ skeletons, directly impacts the protein–flavonoid binding capacity, which was successfully used to understand the anti-inflammatory and anti-diabetic properties of flavonoids [65]. Affinity studies focused on flavonoids and tyrosinase revealed that the hyperpigmentation of skin was reduced due to the hydroxyl groups of flavonoids [66]. Hence, the structure–function relationships of flavonoids upon disease treatment is a topic of interest for researchers. Additional *in silico* and docking studies are necessary to validate these research hypotheses. 

The therapeutic effects of quercetin in age-related conditions were observed *in vitro* and *in vivo* when the concentration used varied between 200 and 1.200 mg for up to 12 weeks. Studies suggest that after quercetin’s metabolism in the gut, there are liberated phenolic acids possessing anti-proliferative and anti-inflammatory capacity by caspase and MMP-7 inhibition, positively modifying the P13K/AKT/MAPK pathway [68,69]. Additional studies confirm the nutraceutical potential of both quercetin and catechin derivatives, which were included in senescence-associated drug formulas to improve the lifespan *in vivo* on mice and humans or to regulate and improve mitochondrial and brain function [70]. Myricetin, kaempferol, and epigallocatechin gallate, along with other flavonoids extracted from plants, were studied simultaneously *in vitro* and *in vivo* on *Caenorhabditis elegans* (*C. elegans*) to demonstrate their dose-dependent effects against Alzheimer’s, cardiovascular diseases, and cancer or in terms of lifespan extension [71,72,73,74]. Various plant parts are rich in flavonoids, which are responsible for skin anti-aging. Specifically, quercetin, isoquercetin, rutin, and epicatechin derivatives have antioxidant properties, thus being able to enhance collagen synthesis, scavenge ROS, and inhibit hyaluronidase, elastase, and collagenase or MMP on skin fibroblasts, besides other anti-aging properties [75,76]. Furthermore, positive effects on UV-induced skin aging were observed on fibroblasts *in vitro* or on mice when apigenin, luteolin, and kaempferol were used as anti-aging agents. The previously mentioned compounds downregulated the MAPK and SASP pathways’ activity. Consequently, the levels of ROS, MMP (MMP-1 and MMP-3), and interleukins (IL-1α, IL-1β, IL-6, IL-8) decreased [77]. 

Anthocyanins are frequently found in plants (e.g., petunidin, peonidin, pelargonidin, malvidin, delphinidin, and cyanidin), offering a red, blue, or purple color. Besides their common utilization as dyes, anthocyanins can be used as nutraceuticals thanks to their beneficial contributions to cardiovascular, carcinogenic, and inflammatory conditions as an effect of senescence [78]. Anthocyanin-rich plant extracts are able to protect against neurodegenerative disorders as an effect of aging. The mechanisms behind this nutraceutical effect are attributed to the antioxidant potential of anthocyanins, which minimizes the synthesis of pro-inflammatory cytokines IL-1β and TNF-α or COX-2 and caspase-3 within the AKT pathway. The anti-aging effect on the nervous system due to anthocyanin administration depends on the anthocyanins’ source and dose. In this regard, concentrations ranging between 4 and 130 mg/kg/day prevented neuronal cells’ apoptosis and improved the spatial memory and learning capacity [79]. 

## 3. Anti-Aging Effects of Flavonoids

The anti-aging effects of various flavonoid-rich plant extracts will be briefly summarized in this section. The phytochemical composition and the nutraceutical effects that are useful in the treatment or prevention of aging-associated conditions will be emphasized as well, according to the available data from the literature. 

### 3.1. Flavonoids from Plants against Skin Aging

Goutweed (*Aegopodium podagraria* L.), Asiatic pennywort (*Centella asiatica* L.), and spignel (*Meum athamanticum* L.) were subjected to ultrasound-assisted extraction with an aqueous glycerol solution (20% *v*/*v*) as an extraction solvent. The richest source of flavonoids was goutweed, whereas the lowest content was determined for spignel. *In vitro* assays on cell lines demonstrated the cytocompatibility of the extracts with keratinocytes and fibroblasts when the evaluated concentrations of the extracts ranged between 0.50% and 10%. Hence, the authors reported that a 5% extract of *Aegopodium podagraria* L. improved cells’ proliferation and determined inhibition ratios of 80% for elastase and 70% for collagenase, being helpful in skin aging conditions [80]. 

Dragon fruit (*Hylocereus undatus* L.) skin extracts were analyzed for their bioactive composition and anti-aging effects. Maceration with ethanol 70% (*v*/*v*) was carried out in order to obtain phytochemical-rich products. The preliminary qualitative evaluation suggested that flavonoids with antioxidant properties were one of the most abundant bioactive classes in the extract. The authors evaluated the radical scavenging properties of the concentrated extract by comparing its potential with that of kaempferol 3-*O*-rutinoside as a positive reference control. In the DPPH assay, the IC_50_ values for the control were lower, as expected (83.30 µg/mL), and higher for the dragon fruit peel extract (164.98 µg/mL). The same trend was observed for the antagonistic activity of the samples against tyrosinase. An IC_50_ value of 88.46 µg/mL was determined for the plant extract, while, for the positive control, the calculated concentration was 59.34 µg/mL. The anti-aging effect of an extract’s compounds is directly affected by the concentration used [81]. The ethanolic extracts of yellow, red, and purple passion fruit (*Passiflora edulis* L.) peels were studied for their anti-aging effects on the skin. Concentrations ranging between 0.05% and 0.25% were included in gel formulations. The results of *in vivo* tests suggested that the purple passion fruit peel extract was the most suitable for gel formulations, the anti-aging effect being dose-dependent. Hence, after constant administration (e.g., for 28 days, twice a day), skin elasticity was improved with 30.08%, along with a moisture recovery rate of 32.74% and a melanin recovery rate of 20.11% [82]. Tyrosinase and esterase inhibition were tested as well for black bean (*Phaseolus vulgaris* L.) coat extracts obtained by different methods. Conventional extraction with water or water and ethanol 1:1 (*v*/*v*), at 40 °C and 150 rpm for 4 h, or supercritical CO_2_ extraction with or without 10% co-solvent (water or water/ethanol), at 300 Barr and 60 °C, was carried out. The subsequent extracts were characterized regarding their phytochemical compositions and anti-aging properties based on the antioxidant properties or skin-related aging enzymes. The reported data suggested that the extraction solvent composed of water and ethanol was more efficient for anthocyanins’ extraction by conventional protocols (7.30 mg cyanidin 3-glucoside equivalents/g coat) compared to supercritical CO_2_ (5.87 mg cyanidin 3-glucoside equivalents/g coat). The qualitative evaluation of the bioactives from the extract by liquid chromatography–mass spectrometry (LC-MS) revealed that myricetin, catechin, cyanidin, malvidin, delphinidin, petunidin, and quercetin derivatives were identified. Therefore, after analyzing the functional characteristics of the extracts, the results showed that the purified extract obtained by conventional extraction had enhanced DPPH (IC_50_ = 0.12 mg/mL) and ABTS (IC_50_ = 0.23 mg/mL) scavenging potential. The extraction method followed by purification was not significant for the anti-tyrosinase assay (IC_50_ = 0.14–0.16 mg/mL). A lower IC_50_ value was associated with the purified conventional extract (IC_50_ = 0.005 mg/mL) when esterase was used [83]. 

Aloe species contain two specific flavonoids, namely orientin and isovitexin, which were able to inhibit the hyaluronidase enzyme with major impacts on skin health and inflammatory reactions [84]. Sweet cherry (*Prunus avium* L.) stems were studied regarding their anti-aging effects associated with their bioactive content following supercritical extraction protocols with CO_2_ (150 Barr, 40 °C, 1 h) and water (20 Barr, 150 °C, 3 Hz, 30 min). These processes allowed the separation of epicatechin, quercetin, rutin, kaempferol, isorhamnetin, and naringin derivatives. The inhibition ratios calculated for elastase (164.11%) and hyaluronidase (90.80%) were significantly different from those of the controls, whereas a non-significant result was determined for the sweet cherry supercritical CO_2_ extract’s inhibition against collagenase and tyrosinase [85].

Various citrus species have been studied in the literature for their anti-aging potential. Specifically, a lemongrass leaf (*Chymbopogon citratus* L.) extract was tested by Ramadhani et al. [86] to be used afterwards for an anti-aging cream formula. Specific parameters such as moisture, smoothness, pore size, the number of spots, and wrinkles were evaluated. After 4 weeks of treatment, the experimental data showed that the lemongrass leaf-based cream increased the moisture content from 2.48% to 14.94%. The same trend was observed for the smoothness level, which increased from 4.19% to 20.42%. Moreover, the same product designed by the previously mentioned authors reduced the pore size, wrinkles, and spots on the skin at the end of the treatment period. Such effects can be associated with the antioxidants originating from the lemongrass leaf extract on the skin tissue and cells. Kim et al. [87] demonstrated that a lemongrass extract produced with ethyl acetate had superior anti-aging properties on the skin. Using a concentration of 50 µg/mL, the authors reported inhibition ratios of 60.78% for elastase and 75.06% for collagenase. Moreover, the nitric oxide and DPPH scavenging ratios of 41.08% or 56.06% confirmed the antioxidant potential of the analyzed extract, which was rich in catechin, luteolin, and isovitexin. Such positive antiradical effects could be associated, as well, with quercetin, kaempferol, and apigenin from the aerial parts of the same species, as reported by some authors [88,89]. Grapefruit peels (*Citrus maxima* Merr.) were macerated for 5 days with ethanol to extract the bioactive compounds. The resulting extract possessed antioxidant activity, being able to scavenge DPPH by 75.28% at a concentration of 40 ppm. The grapefruit peel extract was then included in a gel formulation (1–2%) with sodium carboxymethyl cellulose and glycerin to test its anti-aging potential on the skin. The designed gel could be considered a nutraceutical formula because it improved the moisture content and smoothness of the skin and reduced the pore size, hyperpigmentation, and wrinkles after 4 weeks of treatment [90]. From *Citrus medica* L., known as finger citron, bioactive compounds were extracted with methanol 85% (*v*/*v*) at 0.20 MPa and 90 °C for 120 min; then, after purification and LC-MS analysis, its abundance of hesperidin and naringin derivatives was reported. These flavonoids within the analyzed extract inhibited DPPH and ABTS by 90% or 100% at a concentration of 1 mg/mL. The purified extract of finger citron extended the lifespan of *C. elegans* worms by 31.26% when a concentration of 200 µg/mL was used for *in vivo* tests. The same level of extract improved the stress resistance of the tested worms by 33.33%, which could be correlated with the intensive enzymatic activity in the cells, especially SOD and catalase (CAT) [91]. In another study conducted by Chen at al. [92], finger citron flavonoids were extracted by continuous phase transition equipment. The statistical optimization experiments revealed that the ethanol concentration, extraction temperature, and time were significant factors for the targeted response and the high yield of flavonoids in the extract, respectively. Under the optimized combination of factors (85% ethanol and 90 °C extraction temperature at 0.20 MPa for 120 min), the obtained extract, rich in flavonoids, was able to extend the lifespan of *C. elegans* by 14.94% when a concentration of 200 µg/mL was used. Thus, the authors demonstrated that this extract was able to improve the physical resistance *in vivo*. These experimental data confirm the *in vivo* anti-aging potential of flavonoid-rich extracts based on finger citron. 

British yellowhead flowers (*Inula britannica* L.) were used to extract the bioactive compounds in boiling water (100 °C for 1 h). Then, a flavonoid-rich purified phase was obtained, which was further used for *in vivo* tests on mice to evaluate the anti-aging properties. The authors tested the purified plant extract via administration for 6 weeks to mice in various doses ranging between 0.50 and 400 mg/kg BW. Low and medium doses seemed to improve significantly the enzymatic activity of SOD, CAT, and glutathione peroxidase at the skin level, decreasing simultaneously the concentration of malondialdehyde. The mechanisms behind these outcomes were related to the increased expression level of SRT1, correlated with a decrease in the p16 and p21 proteins, which are directly responsible for cellular senescence. It should be mentioned that the observed nutraceutical effect is dependent on the administered concentration, as an increased dose may lead to contrasting side effects [93]. 

Ethanol 70% (*v*/*v*) and maceration were applied to *Ageratum conyzoides* L. (common name: billygoat weed) to efficiently extract the phytochemicals. After preliminary characterization, the obtained extract was a combination of phenols and flavonoids with anti-aging effects. Apart from the DPPH scavenging rate (IC_50_ = 80.70 µg/mL), the mixture of compounds from the analyzed plant extract had anti-aging effects on the skin based on collagenase (IC_50_ = 55.07 µg/mL) and elastase (IC_50_ = 45.35 µg/mL) inhibition [94]. The same extraction protocol was followed for *Hibiscus sabdariffa* L. (Roselle) phytochemicals’ extraction. Using ultra-high-performance liquid chromatography–mass spectrometry analysis (UHPLC-MS), it was demonstrated that myricetin was the main flavonoid within the analyzed extract. Its inhibitory potential against skin-aging associated enzymes was evaluated, concluding that the extract had antioxidant activity against DPPH (76.79%) and ABTS (34.37%), as well as anti-collagenase (IC_50_ = 750.33 µg/mL), anti-elastase (IC_50_ = 103.83 µg/mL), and anti-hyaluronidase (IC_50_ = 619.43 µg/mL) properties [95]. Roselle calyx extract produced by maceration with ethanol 70% (*v*/*v*) for 72 h was included in various cream formulations (0.50–2%) to evaluate its anti-aging effects on the skin. Based on specific scales, the moisture, smoothness, pore and spot size, and wrinkles were evaluated after guinea pigs’ skin exposure to UV light for 5 h. The reported data suggest that, after irradiation, the cream with 2% Roselle extract was the most efficient formula, being responsible for improved moisture and evenness. Other positive effects of the designed cream were related to the ability to reduce wrinkles, spots, and the pore size [96]. 

*Mucuna pruriens* L. seed extract, at a concentration of 1 mg/mL, led to the inhibition of collagenase (55.33%), elastase (32.12%), and hyaluronidase (81.13%), yielding data that confirmed its anti-aging properties on the skin [97]. *Perilla frutescens* seed extract was efficient in minimizing skin aging *in vitro*, as reported by Mungmai et al. [98], this conclusion being related to the antioxidant compounds’ ability to decrease the collagenase activity by up to 82% at a concentration of 400 µg/mL and to exert anti-melanogenesis effects at a concentration of 40 µg/mL. Such observations confirm its potential use as a nutraceutical agent to overcome skin wrinkles or hyperpigmentation. 

For parsley (*Petroselinum sativum* L.), the bioactive composition of the ultrasound-assisted ethanolic extract was characterized in terms of its flavonoid content, indicating that gallocatechin, isorhamnetin, quercetin, apigenin, and kaempferol were identified. Its biocompatibility with human keratinocytes was demonstrated considering parsley extract concentrations ranging between 0.10 and 100 µg/mL. The ROS neutralization seemed to be improved after the preliminary cultivation of the cells with the analyzed extract, possibly due to the protective effects of the flavonoids on the cellular components, especially on mitochondria. The inhibitory effect of the ethanolic parsley extract against elastase was superior (IC_50_ = 3 µg/mL) compared to the result obtained for kojic acid (IC_50_ = 21.60 µg/mL). A similar trend was observed for the ethanolic extract on tyrosinase (IC_50_ = 2.03 µg/mL), while, for kojic acid, an increased amount was necessary for the same effect (IC_50_ = 9 µg/mL). The IC_50_ values for collagenase (IC_50_ = 10.18 µg/mL) and hyaluronidase (IC_50_ = 12.54 µg/mL) were significantly lower than the ones calculated for the positive controls [99]. 

Luteolin derivatives or native quercetin and kaempferol were found in Ulrica species, namely *Ulrica doica* L. and *Ulrica thunbergiana* L. extracts. Interestingly, at well-known doses, such extracts exert antioxidant effects *in vivo* (50–100 mg/kg, for 14 days) by modifying the peroxidase, dismutase, and reductase activity, respectively, possessing anti-aging effects on the skin when 0.1% or 1% extract was administered for 10 weeks. The therapeutic effects were demonstrated via wrinkle reductions and skin moisture improvements [100].

*Nelumbo nucifera* L. flowers, leaves, and seeds were tested *in vitro* for skin anti-aging using specific enzymes such as collagenase, elastase, hyaluronidase, and tyrosinase. LC-MS revealed numerous anthocyanins in the samples (malvidin, delphinidin, cyanidin, petunidin hexosides, and pentosides), along with kaempferol, quercetin, isorhamnetin, myricetin, apigenin, epicatechin, and gallocatechin in isomeric forms. The results of the anti-aging-associated enzyme analysis revealed that the lotus flower extract had superior inhibitory effects on collagenase (IC_50_ = 1.30 µg/mL), elastase (IC_50_ = 10.50 µg/mL), tyrosinase (IC_50_ = 14.60 µg/mL), and hyaluronidase (IC_50_ = 12 µg/mL) [101]. Furthermore, the lotus stamen extract was employed in the ultrasound-assisted extraction of flavonoids with 90% (*v*/*v*) ethanol, for 45 min, at 45 °C and 45 kHz. The compositional characterization revealed the presence of myricetin-3-*O*-glucoside, quercetin-3-*O*-glucuronic acid, kaempferol-3-*O*-glucuronic acid, and isorhamnetin-3-*O*-glucoside as major flavonoid representatives. *In vivo* tests on *Saccharomyces cerevisiae* emphasized the extract’s capacity to significantly expand the lifespan by 7.66 days, compared to the control yeast with a decreased lifespan of 5.75 days. The lifespan extension could be attributed to the protective effect of flavonoids on the cells—specifically on the mitochondrial integrity, which was improved after 10 days. The extract acts on the central metabolism, supporting its normal activity over time. The anti-aging properties were demonstrated as well by the improved activity of oxidative enzymes, especially SOD, involved in cellular senescence [102]. 

*Persicaria minor* L. extracts were characterized by superior antioxidant potential, determined by quercetin, myricetin, apigenin, isorhamnetin, and catechin derivatives, able to inhibit SOD, CAT, glutathione peroxidase, or elastase, thus being an important skin anti-aging agent [103]. 

The skin anti-senescent effects of *Trichosanthes kirilowii* L. leaf extract were studied by Zhang et al. [104], emphasizing the capacity of individual compounds (e.g., apigenin, quercetin, and luteolin) within the extract to overcome skin aging by inhibiting melanin and tyrosinase production. 

*Dracocephalum moldavica* L. extract’s effect on skin was evaluated by Wandrey et al. [105]. Due to the glucuronides identified in the extract, especially apigenin glucuronide, at a concentration of 0.50% extract used *in vitro* on cell cultures, it was observed to exhibit a stimulatory effect on the AMPK pathway, thus increasing the antioxidant cellular response, as well as causing improved collagen synthesis, both preventing skin aging. 

The flavonoids within *Moringa oleifera* L. leaf extract exhibited inhibitory effects against elastase (IC_50_ = 253.95 µg/mL) and collagenase (IC_50_ = 355.58 µg/mL). Interestingly, molecular docking studies justified the inhibitory activity of the compounds from the extract, namely apigenin glucoside, quercetin-3-*O*-glucoside, kaempferol rhamnoside, kaempferol-3-*O*-rutinoside, kaempferol-3-*O*-glucoside, and isorhamnetin-3-*O*-glucoside, via their binding capacity to hyaluronidase, therefore decreasing its activity [106]. The SOD, tyrosinase, and elastase inhibition determined for *Moringa oleifera* L. powder was screened *in vitro*. For SOD, concentrations between 1.56 and 3.13 mg/mL had a positive effect, whereas tyrosinase and elastase were inhibited at up to 25 mg/mL [107]. Furthermore, the anti-aging effect of this extract on the brain was justified by the reduction of lipid peroxidation. Therefore, the administered dose is a crucial factor because the plant extract’s cytocompatibility differs depending on the tested model. In particular, when the extract was administered *in vitro*, a concentration of 50 µg/mL affected cells’ viability, whereas *in vivo* studies suggested that 20 g/kg did not exert negative effects [108]. 

Isoquercitin was successfully isolated from *Ephedra alata* Decne following a statistically optimized ultrasound-assisted extraction process. This compound has anti-aging potential due to its ability to differently inhibit enzymes associated with skin senescence and inflammation, such as collagenase (87.31%), elastase (88.93%), and hyaluronidase (98.83%) [109]. Luteolin extracted from *Rosenda luteola* L. leaves had anti-inflammatory properties, being able to reduce skin irritation in volunteers. Depending on its origin, luteolin from *Bryophyllum pinnatum* (Lam.) was efficient in edema reduction in combination with other flavonoids such as rutin, quercetin, or luteolin derivatives [110]. Bamboo leaf (*Bambusa vulgaris* L.) extract containing orientin, isoorientin, vitexin, and isovitexin was used as an ingredient (2.50–5%) for an emulsion, which demonstrated anti-aging effects on skin *in vivo* on mice subjected to UV radiation, but additional studies are necessary on this subject to confirm the relationship between bamboo leaf flavonoids and the MAPK pathway [111]. *Eutrema japonicum* (Miq.) Koidz., known as Japanese horseradish, was used to extract various phytochemicals from the leaves, roots, and flowers. The results suggested that the flowers were abundant in flavonoids, namely luteolin, apigenin, quercetin, isorhamnetin, and isovitexin, with skin anti-aging effects demonstrated in terms of *in vitro* anti-collagenase (78.42–93.34%), anti-elastase (84.90–90.18%), and anti-hyaluronidase (13.46–47.32%) activity, which was highly dependent on the botanical part analyzed and the plant’s maturity [112]. 

The flavonoids with skin anti-aging potential extracted from plant species are summarized in Table 1.

### 3.2. Flavonoids from Plants against Brain Aging

Brain aging processes could be delayed as a consequence of flavonoid-rich plant extracts’ administration, as some *in vitro* and *in vivo* experimental studies and review papers, respectively, suggest. 

A rabbiteye blueberry (*Vaccinium virgatum* L.) leaf extract rich in flavonoids, especially quercetin-3-*O*-galactoside, quercetin-3-*O*-rutinoside, quercetin-3-*O*-glucoside, and kaempferol-3-*O*-glucoside, was included in a nanoemulsion that was then administered to rats in high doses (40 mg/kg BW) to emphasize the nutraceutical effect related to the neuroprotective properties by modifying the dopamine levels. Additionally, hepatic function was significantly improved due to SOD, glutathione peroxidase, and CAT’s activity, after the administration of the formulated nanoemulsion [113]. *Persicaria minor* L. extract at concentrations of 250 mg, administered twice a day for six months to patients aged between 60 and 75 years, improved cognitive function by facilitating novel neuronal lineages. Such positive effects were associated with quercetin 3-*O*-glucuronide, a flavonoid that is abundant in the commercially available extract [114]. A positive effect was also demonstrated *in vivo* on rats. Specifically, an aqueous extract of this plant species dosed at 200 or 300 mg/kg was used to treat rats with dementia or Alzheimer’s for 14 days, concluding that the previously mentioned doses ameliorated memory-related problems [115]. 

The antioxidant compounds within an okra extract (*Abelmoschus esculentus* L.), such as quercetin, isoquercetin, quercetin-3-*O*-rubinobioside, and myricetin [116], facilitated neuronal cell proliferation *in vitro* and reduced the senescence-induced effects at a concentration of 75 µg/mL by alleviating the ROS levels [117]. 

Furthermore, the anti-aging effect of *Moringa oleifera* L. extract on the brain was justified by the reduction of lipid peroxidation due to the abundance of apigenin, kaempferol, quercetin, or isorhamnetin derivatives. Quercetin, catechins, and epicatechins’ neuroprotective properties were confirmed as well by other authors [118]. It is important to mention that the administered dose is crucial, because this plant extract’s cytocompatibility differs depending on the tested model. In particular, when the extract was administered in vitro, a concentration of 50 µg/mL affected cells’ viability, whereas *in vivo* studies suggested that 20 mg/kg did not exert negative effects [108]. 

The main findings related to plant flavonoids with brain anti-aging properties are briefly summarized in Table 2.

### 3.3. Flavonoids from Plants with Anti-Inflammatory Potential

Some age-related disorders’ mechanisms have been explained in the literature in order to manage their risks and incidence. Consequently, it seems that the anti-inflammatory properties of flavonoids are related to specific enzymes’ inhibition, namely COXs and lipoxygenases, and their in-depth effects on interleukin production are correlated with the minimization of pro-inflammatory reactions [18]. The expression of COX-2 within the MAPK pathway, along with nitric oxide synthase, was positively modified by naringenin, quercetin, myricetin, luteolin, or kaempferol [118,119]. These mechanisms clarify the anti-inflammatory properties of specific flavonoids with anti-aging potential [120]. Orientin and isovitexin, specific flavonoids of Aloe species, exhibit anti-inflammatory potential *in vitro* and/or *in vivo* by positively modifying the levels of cytokines and C-reactive protein [84]. The anti-inflammatory potential of a lotus flower (*Nelumbo nucifera* L.) extract was demonstrated *in vitro* on lipoxygenase and COX (IC_50_ = 9.98 µg/mL for COX-1; IC_50_ = 13.26 µg/mL for COX-2) inhibition, respectively [101].

The constant administration of lemongrass (*Cymbopogon citratus* L.) tea (10% or 20%) was beneficial in terms of the anti-inflammatory effects of the plant species on rats [121]. *Ephendrae herba* L. is a rich source of various flavonoids, such as rutin, catechins and epicatechins, naringenin, luteolin, quercetin, hesperidin, and kaempferol, which may exert anti-inflammatory effects *in vitro* at 50 or 100 µg/mL [122]. 

The anti-inflammatory effects of *Anchusa azurea* Mill. roots’ ethanolic extract were observed *in vivo* at a concentration of 200 or 400 mg/kg. Catechin and quercetin-3-rhamnoside were identified in the extract by LC-MS, with these flavonoids being responsible for the evaluated properties [123]. Lower concentrations of the ethanolic extract, namely 50 or 100 mg/kg, improved brain function [124]. 

Heliotropium varieties’ pharmacological properties were summarized by Fayed et al. [125] to highlight the importance of *in vitro* edema models and anti-inflammatory reactions and the influence of the extraction solvents used on the expected results. The above-mentioned review paper reported that the hexane fractions of a *Heliotropium strigosum* L. extract rich in quercetin exhibited edema inhibition ratios between 70.66% and 73.33% depending on the particularities of the simulated model, whereas the maximum inhibition of 80% was calculated for an extract of *Heliotropium indicum* L. when a dose of 150 mg/kg BW was tested. Quercetin, myricetin, and kaempferol were the main flavonoids identified in *Heliotropium crispum* L. when methanol, ethyl acetate, or chloroform was used as an extraction solvent [126]. Such bioactives within the extracts may be beneficial for the anti-senescence properties on brain cells.

The flavonoids that may have brain anti-inflammatory potential, based on the cited literature, are summarized in Table 3. 

### 3.4. Flavonoids from Plants with Cardio-Protective Effects

An imbalanced diet and unhealthy lifestyle affect the human body’s functions. Hence, free radicals are liberated or produced in the body, which can act on low-density lipoproteins (LDL) by oxidizing them and finally generate fat deposits on the blood vessels. Fortunately, thanks to their free radical scavenging power, flavonoids may exert anti-atherosclerotic properties that contribute to cardio-protective and anti-aging effects [18]. The ROS and nitric oxide pathways were influenced by naringenin, which minimized the free radical production. As a consequence, vasodilation correlated with improved circulatory function was observed, due to caspase-3 enzyme activity [120]. Quercetin and its derivatives possess cardio-protective properties by reducing LDL cholesterol oxidative reactions [127]. Similar mechanisms were observed for other flavonoids, namely kaempferol, catechins, and epicatechins [118]. Additional *in vitro* studies suggest that the anti-aging effects on endothelial cells can be associated with quercetin (25 µM), kaempferol (50–100 mg/kg, 4-week treatment), or luteolin (0.50–2 µM) administration, which positively modifies the pro-inflammatory cytokines, nitric oxide, or SOD activity [128]. Isorhamnetin-3-*O*-rutinoside was tested for the same effect on endothelial cells *in vitro* using the EA.hy 926 cell line, demonstrating that a 40 µM dose of the previously mentioned flavonoid increased the activity of nitric oxide synthase in endothelial cells [129]. Human umbilical vein endothelial cells (HUVECs) were used as well for *in vitro* tests to emphasize the binding potential of kaempferol (10 µM) with vascular endothelial growth factor to promote blood vessel reconstruction. Supplementary *in vivo* tests on zebra fish or rats demonstrated that this health-promoting effect was associated with the endothelial nitric oxide levels, directly involved in vasodilation [130]. 

Quercetin and kaempferol originating from *Lens culinaris* Medik have anticoagulant potential [131] and could be used as nutraceuticals for cardiovascular diseases [132]. Additionally, their cholesterol-lowering properties were attributed to catechin, gallocatechin, cyanidin, or delphinidin when lentil extracts were used for *in vivo* tests, taking into consideration doses of 100, 200, or 400 mg/kg [133]. An anti-hypercholesterolemic effect was attributed also to apigenin derivatives from *Ajuga iva* L. [131]. Various flavonoids from this plant species (i.e., myricetin, luteolin, quercetin, rhamnetin, apigenin derivatives, naringin and naringenin, catechins, and epicatechins) had a significant anti-hypertensive effect on rats at 500 mg/kg after 10 days of administration [134,135]. The toxicity of *Ajuga iva* L. aqueous or methanolic extracts was tested using *in vitro* models, concluding that the oral administration of the extracts was safe from 100 mg/kg to 2000 mg/kg, whereas adverse effects were observed after interperitoneal injection with 3600 mg/kg or higher doses [136]. Luteolin and apigenin from lemongrass (*Cymbopogon citratus* L.) had cardio-protective effects *in vivo* (10 µM) due to their vasodilation properties [131]. The research paper published by Wang et al. [137] confirms the positive effect on chronic myocardial infarction. In particular, extract doses of 30 or 50 mg/kg BW, administered daily for 4 weeks, downregulated the levels of TNF-α, IL-1β, IL-6, and caspase-3, thus improving cardiac function due to the rutin, quercetin, kaempferol, naringenin, and hesperidin within the *Anchusa italica* Retz. (*Anchusa azurea* Mill.) ethanolic extract [131]. The cardio-protective effect of the aqueous extract from *Heliotropium taltalense* L. was demonstrated *in vivo* by Barrientos et al. [138], suggesting that, starting from 50 µg/mL, heart contractions were reduced, along with a hypotensive effect at 100 µg/mL as a consequence of the nitric oxide synthase activity. These results were reported for an aqueous extract with antioxidant potential containing naringenin and quercetin [131]. 

Quercetin, kaempferol, luteolin, rutin, and apigenin derivatives from *Trichosanthes kirilowii* L. leaf extract [139] possess anti-aging properties, as studied by He et al. [140]. The authors suggest that the flavonoids within the extract produce vasodilation in the circulatory system, especially in the blood vessels responsible for proper cardiac function. This outcome could be attributed to the previously mentioned phytochemicals that increase the phosphorylation in specific signaling pathways, such as P13K/AKT. Luteolin, kaempferol, naringenin, quercetin, and isorhamnetin’s anti-aging effects were studied *in silico* in order to enhance the knowledge about their mechanisms of action. The results highlighted that quercetin, kaempferol, and luteolin may have an improved interaction with proteins associated with the aging process, namely AKT1, HSP90AA1, and IL-6. The above-mentioned phytochemicals can modify the aging proteins’ metabolic pathways and therefore delay the senescence-associated side effects [141].

*Ephendrae herba* L. could be used as a cardio-protective agent. Rutin, epicatechins, quercetin, hesperidin, and kaempferol [131] exert anti-hypertensive effects *in vivo* at 0.10–10 mg/kg [122]. Isorhamnetin with hypotensive potential, originating from *Corchorus olitorius* L. leaf extracts, was studied by Kumari et al. [142], concluding that the previously mentioned flavonoid can decrease the ACE activity associated with hypertension and heart disease. 

Table 4 presents a summary of the information regarding the cardio-protective effects of flavonoids from different plant sources.

### 3.5. Flavonoids from Plants with Anti-Diabetic Properties

The glycosylated forms of quercetin are frequently found in plants, but their bioavailability is limited unless they are subjected to structural changes involving targeted enzymatic reactions to increase quercetin’s absorption. Studies suggest that constant quercetin administration is beneficial in diabetes treatment. The anti-diabetic effects are linked to C-reactive protein and TNF-α [143]. Therefore, the anti-diabetic effect of lemongrass (*Cymbopogon citratus* L.) methanolic extract was attributed to the abundance of rutin in the extract, which was able to downregulate the TNF-α pro-inflammatory cytokine and other specific genes involved in diabetes symptoms when doses of 100 or 400 mg/kg or 1.50 mg/100 g were used on *in vitro* models [144,145]. 

*Heliotropium procubens* L. flavonoids, mainly kaempferol, catechin derivatives, luteolin, and naringenin-7-*O*-glucosides, were identified in the methanolic or water extracts of this plant, with their anti-aging effects being related to the anti-diabetic properties exhibited via α-amylase and α-glucosidase inhibition [146]. 

Some nutraceutical flavonoids (glycosylated forms of kaempferol and quercetin or native luteolin and apigenin) were identified in a horsetail (*Equisetum arvense* L.) extract. Rats treated with doses of 100 mg/kg for 6 weeks had improved glucose and insulin tolerance, demonstrating the extract’s anti-diabetic potential as an effect of SIRT1 metabolic pathway activation along with its specific proteins [147,148]. Furthermore, blood glucose levels were reduced *in vivo* in diabetic mice whose diet was supplemented with an okra-based (*Abelmoschus esculentus* L.) beverage dosed at 0.52 mL/day. The hypoglycemic effect was time-dependent, with significant changes being observed after 12 days of consumption [149]. The anti-diabetic effects of kaempferol, quercetin, luteolin, naringin, naringenin, quercetrin, and rutin from *Corchorus olitorius* L. were described by Ben Yakoub et al. [150]. The influence of the plants’ cultivation conditions on the quercetin and kaempferol glucoside content was reported by Guzzetti et al. [151], emphasizing the impact of agricultural practices on the oxidative enzymes’ inhibition, namely SOD and CAT, mainly responsible for ROS production *in vitro* and *in vivo*, as well as for aging-related conditions. 

Indian almond (*Terminalia catappa* Linn.) leaf extract, abundant in orientin and vitexin, was used in high-glucose-induced and obese *C. elegans*. The extract must be used carefully because it can induce toxicity *in vivo* when the concentration range is between 50 and 400 µg/mL. Meanwhile, non-toxic concentrations were established between 6.25 and 25 µg/mL [152]. *C. elegans* was used to test the anti-diabetic effects of cyanidin and peonidin glucosides, as well as malvidin galactoside, from purple wheat (*Triticum* sp.). These phytochemical compounds, dosed at 100 µg/mL, inhibited the IGF-1 pathway, positively modifying specific insulin receptors (DAF-2). Furthermore, the purple wheat anthocyanins were able to improve the stress resistance *in vivo* [153]. 

The anti-diabetic properties of the flavonoids extracted from the plant species discussed in this section are summarized in Table 5.

### 3.6. Flavonoids from Plants with Anticarcinogenic Effects

Mitochondrial dysfunction leads to cellular ROS accumulation, which is a precursor for degenerative age-related diseases and cancers. Malignant cell proliferation can be modified by flavonoids, which determine apoptosis by triggering the production of caspase-3, caspase-6, caspase-7, and caspase-9 by autophagy stimulation through the inhibition of signaling pathways or pro-inflammatory cytokines (TNF-α, IL-1β, IL-6, and IL-8) [154]. For instance, hesperidin and naringenin contribute to anti-aging effects by inducing cancerous cells’ apoptosis through the activation of the caspase-3 signaling pathway and the regulation of mitochondrial function [120]. 

Flavonoids have been intensively studied for their potential to inhibit cancerous cells’ proliferation. The positive outcomes observed *in vitro* are dose-dependent. In particular, epigallocatechin gallate was used in breast (1–40 µM), lung (5–20 µM), prostate (1 mg/3 doses per week), colorectal (1–50 µM), and gastric (20–100 µM) cancer treatment. Doses of quercetin were efficient in cervical (110.38 µM) or breast cancer (1–200 µM) treatment. Moreover, luteolin doses of 10–30 mg/kg or ranging between 20 and 100 µM inhibited lung cancer and colon cancer cells’ proliferation, respectively. The proliferation of malignant cells developed in prostate, colorectal, or gastric cancer could be reduced by apigenin administration considering doses of 20 or 50 µg/mouse or 20 µg/mL, respectively [155]. Green tea epigallocatechin gallate doses between 5 and 50 µM/mL were beneficial against breast cancer cells *in vitro*, but a decisive factor in this therapeutic effect is related to epigallocatechin gallate’s final concentration in the blood and plasma [156]. Luteolin derivatives or native quercetin, kaempferol, and isovitexin were found in *Ulrica doica* L. and *Ulrica thunbergiana* L. extracts [100]. Interestingly, in well-known doses (10 or 20 mg/kg for 28 days), such extracts exert anti-tumor activity by reducing the tumor size and proliferation, along with an increment in cancerous cells’ apoptosis [118].

*Lens culinaris* Medik was subjected to solid–liquid extraction with acetonitrile and water (70:30 *v*/*v*) to emphasize the abundance of kaempferol 3-*O*-arabinoside and apigenin glucoside after LC-MS analysis. Furthermore, the extract was screened for its adjuvant effect when it was simultaneously used with chemotherapeutics. The data reported by Di Turi et al. [157] suggest that the positive effects of lentil extracts are dose-dependent, as concentrations between 0.10 and 5 mg/mL impacted differently the proliferation of osteoblasts or muscular fiber function. Lentil bioactives lead to the apoptosis of breast cancer cells due to kaempferol, quercetin, and myricetin [158]. 

The *in vitro* properties on prostate cancer cells were tested, concluding that the targeted therapeutic effect was dose-dependent in the concentration range of 30–1000 µg/mL when lemongrass (*Cymbopogon citratus* L.) extract was used [121]. In another *in vivo* study, doses of 16 mg/kg of this plant extract were tested to demonstrate its efficiency for colon cancerous cells’ apoptosis as an effect of luteolin from the ethanolic extract [144,145]. Luteolin was also identified in horsetail (*Equisetum arvense* L.) extracts, along with apigenin, kaempferol-3-*O*-glucoside, quercetin, and quercetin-3-*O*-glucoside, as major flavonoid representatives. These compounds exhibited inhibitory effects against pancreatic and ovarian cancer when *in vitro* tests involved extract concentrations of 100 µg/mL to 1000 µg/mL [159,160]. Pancreatic cancer cells’ apoptosis was observed along with quercetin administration *in vitro* [161]. 

The needles and twigs of *Taxa fauna* L., *Taxa yunnanensis* L., and *Taxa baccata* L. have shown high levels of apigenin with anti-cancer potential. Moreover, luteolin, kaempferol, myricetin, quercetin, and catechin derivatives from *Taxa brevifolia* L. and *Taxa mairei* L. twigs, needles, or leaves were responsible for similar nutraceutical effects [162]. 

*Asplenium nidus* L. was found to be an excellent source of quercetin 7-*O*-rutinoside, demonstrating its anti-proliferative properties in carcinoma and specifically in hepatic cancer [161].

The plant-derived flavonoids with anticarcinogenic effects that were presented in this section are summarized in Table 6. 

## 4. Conclusions and Future Perspectives 

Multiple studies suggest that aging is mainly impacted by RONS, which further modify the DNA and telomere structure. Furthermore, alterations of some aging-associated pathways are responsible for the inflammatory response that triggers the receptors for cancer, skin, myocardial, or brain disorders, as well as diabetes. These mechanisms were briefly explained in this paper. In addition, the effects and applications of specific flavonoids originating from various plant species that are efficient in the prevention or treatment of the previously mentioned conditions were presented.

In conclusion, the exploitation of plants for their richness in phenolic compounds, including flavonoids, is a promising and innovative approach that can facilitate development in the fields of nutraceuticals, cosmeceuticals, and functional foods. 

On one hand, the bioactives’ extraction protocols, as well as their purification and chemical characterization, are crucial steps that must be considered for future studies in the field. On the other hand, the design processes of nutraceutical formulations with diverse applications must take into account the phytochemicals’ enhanced stability according to the desired effects and objectives. 

Nevertheless, in-depth *in vitro*, *in vivo*, and clinical studies are necessary in order to demonstrate or confirm the nutraceutical properties of the flavonoids extracted from sustainable sources. 

## Figures and Tables

**Table 1:** Plant flavonoids with skin anti-aging effects.

Plant Species	Extraction Parameters	Skin Anti-Aging Flavonoid(s)	Assessment Procedure and Dose	Reference(s)
*Aegopodium podagraria* L. extract	Ultrasound-assisted extraction for 48 min; mixture of water and glycerin (80:20).	Improved cell proliferation; collagenase and elastase inhibition.	*In vitro* using commercial kits and doses between 0.50 and 5%.	[80]
*Hylocereus undatus* L. skin extract	Maceration with 70% ethanol.	Antioxidant properties; tyrosinase inhibition.	*In vitro* with testing doses between 3.13 and 100 µg/mL.	[81]
Purple *Passiflora edulis* L. peel extract	Maceration with 70% ethanol for 5 days at room temperature.	Skin elasticity and moisture improvements; melanin recovery.	*In vivo* on white rats, treated with 0.05–0.25% extract in a gel formulation, used twice a day for 28 days.	[82]
*Phaseolus vulgaris* L. coat extract	Conventional extraction with water or water and ethanol (1:1 *v*/*v*) for 4 h at 40 °C; supercritical fluid extraction at 300 Bar, 60 °C with 10% water or ethanol/water (1:1 *v*/*v*) as cosolvents.	Myricetin, catechin, and quercetin derivatives or cyanidin, malvidin, delphinidin, and petunidin derivatives, respectively, with DPPH and ABTS scavenging activity; anti-tyrosinase and anti-elastase properties.	*In vitro*.	[83]
Aloe species	Ethanol, methanol, petroleum ether, chloroform, ethyl acetate, water.	Orientin and isovitexin with anti-hyaluronidase inhibition properties.	Not reported.	[84]
*Prunus avium* L. stems	Ethanol and water in equal ratios at 40 °C for 20 min at 1500 psi; supercritical fluid extraction at 150 Bar, for 1 h, at 40 °C, 22 g/min CO_2_ with 15% ethanol as cosolvent; supercritical water extraction for 30 min at 20 Bar, 150 °C, 3 Hz.	Epicatechin, quercetin, rutin, kaempferol, isorhamnetin, and naringin derivatives with elastase and hyaluronidase inhibition properties.	*In vitro* using extract concentrations between 0.001 and 0.02%.	[85]
*Chymbopogon citratus* L. leaf extract	Not reported.	Skin moisture and smoothness improvements; pore size, wrinkle, and spot reductions.	*In vivo* on volunteers.	[86]
	80% methanol followed by fractionation with n-hexane, ethyl acetate, or butanol.	Antioxidant properties due to catechin, luteolin, and isovitexin; elastase and collagenase inhibition.	*In vitro*.	[87]
*Citrus maxima* Merr. extract	Maceration for 5 days with ethanol.	Hyperpigmentation and wrinkle reduction; improvements in the moisture and smoothness of the skin.	*In vivo* on volunteers treated 2 times a day for 4 weeks with a gel formula containing 1–2% extract.	[90]
*Citrus medica* L. extract	85% ethanol at 0.20 MPa, 90 °C for 2 h.	Hesperidin and naringin derivatives with free radical scavenging potential.	*In vivo* on *C. elegans* using a dose of 200 µg/mL.	[91]
*Inula britannica* L. extract	Boiling in water at 100 °C for 1 h.	Improved the skin enzymes’ activity.	*In vivo* on mice treated for 8 weeks with 100–400 mg/kg.	[93]
*Ageratum conyzoides* L. extract	Maceration in 70% ethanol.	Antioxidant properties; collagenase and elastase inhibition.	*In vitro*.	[94]
*Hibiscus sabdariffa* L. extract	Maceration in 70% ethanol for 24 h.	Antioxidant activity; anti-collagenase, anti-elastase, and anti-hyaluronidase properties.	*In vitro*.	[95]
	Maceration in 70% ethanol for 72 h.	Improved skin moisture and smoothness.	*In vivo* on guinea pigs treated for 4 weeks, twice a day, with a cream formulation containing 0.50–2% extract.	[96]
*Mucuna pruriens* L. extract	Soxhlet extraction with ethanol for 5 h at 80 °C.	Inhibition of collagenase, elastase, and hyaluronidase.	*In vitro* tests using a dose of 1 mg/mL.	[97]
*Petroselinum sativum* L. extract	Ultrasound-assisted extraction with ethanol, water, or mixtures using 30% amplitude, 20 Hz, at 5 °C for 20 min.	Gallocatechin, isorhamnetin, quercetin, apigenin, kaempferol determined elastase, tyrosinase, collagenase, and hyaluronidase inhibition.	*In vitro*.	[99]
*Ulrica doica* L. and *Ulrica thunbergiana* L. extracts	Ethanolic, aqueous extracts, or dichloromethane- or petroleum ether-based extracts.	Luteolin, quercetin, and kaempferol inhibited peroxidases, dismutases, and reductases; wrinkle reduction and skin moisture improvement.	*In vitro*.	[100]
*Nelumbo nucifera* L. extracts	80% methanol.	Malvidin, delphinidin, cyanidin, petunidin hexosides, and pentosides; kaempferol, quercetin, isorhamnetin, myricetin, apigenin, epicatechin, and gallocatechin, respectively, from lotus flower inhibited collagenase, elastase, tyrosinase, and hyaluronidase.	*In vitro*.	[101]
	Ultrasound-assisted extraction for 45 min, at 45 °C, 30 kHz, with 90% ethanol.	Myricetin-3-*O*-glucoside, quercetin-3-*O*-glucuronic acid, kaempferol-3-*O*-glucuronic acid, and isorhamnetin-3-*O*-glucoside had SOD inhibition potential.	*In vitro*.	[102]
*Persicaria minor* L. extract	Not reported.	Quercetin, myricetin, apigenin, isorhamnetin, and catechin derivatives inhibited SOD, CAT, glutathione peroxidase, or elastase.	*In vitro*, *in vivo*, preclinical trials.	[103]
*Trichosanthes kirilowii* L. extract	Ethanol 95% and ethyl acetate in various combinations.	Apigenin, quercetin, and luteolin inhibited melanin and tyrosinase production.	*In vitro*.	[104]
*Dracocephalum moldavica* L. extract	Ethanol 30% for 3 h at 50 °C.	Apigenin glucuronide increased the antioxidant cellular response; improved collagen synthesis.	*In vivo* on *C. elegans*.	[105]
*Moringa oleifera* L. extracts	Ethanol 90% overnight.	Apigenin glucoside, quercetin-3-*O*-glucoside, kaempferol rhamnoside, kaempferol-3-*O*-rutinoside, kaempferol-3-*O*-glucoside, and isorhamnetin-3-*O*-glucoside with anti-elastase, anti-collagenase, or hyaluronidase-binding potential.	*In vitro*.	[106]
	Not reported.	SOD, tyrosinase, and elastase inhibition.	*In vitro* using doses between 1.56 and 25 mg/mL.	[107]
*Ephedra alata* Decne	Ultrasound-assisted extraction for 10 min, 60 °C, 75 W, with ethanol 70%.	Isoquercetin inhibited collagenase, elastase, and hyaluronidase.	*In vitro*.	[109]
*Bambusa vulgaris* L. extract	Not reported.	Orientin, isoorientin, vitexin, and isovitexin with UV protection potential upon skin anti-aging.	*In vivo* on mice treated with an emulsion containing 2.50–5% extract.	[111]
*Eutrema japonicum* (Miq.) Koidz.	Mixture of methanol, acetone, and water in a ratio of 3:1:1, for 30 min at 40 °C.	Luteolin, apigenin, quercetin, isorhamnetin, and isovitexin with anti-collagenase, anti-elastase, and anti-hyaluronidase activity.	*In vitro* using a dose of 100 µg/mL.	[112]

**Table 2:** Plant flavonoids with brain anti-aging effects.

Plant Species	Extraction Parameters	Flavonoid(s) Responsible for Anti-Aging	Assessment Procedure and Dose	Reference(s)
*Vaccinium virgatum* L. leaf extract	Various concentrations of ethanol (30%, 50%, 70%) at 60 °C for 3 h.	Quercetin-3-*O*-galactoside, quercetin-3-*O*-rutinoside, quercetin-3-*O*-glucoside, and kaempferol-3-*O*-glucoside with neuroprotective effects.	*In vivo* in mice treated for 6 weeks with 8 mg/kg or 40 mg/kg with extract or nanoemulsion containing the extract.	[113]
*Persicaria minor* L. extract	Commercial product based on aqueous extract.	Quercetin 3-*O*-glucuronide improved cognitive and neuronal function.	Clinical trial, 250 mg capsule.	[114]
	Commercial product based on aqueous extract.	Dementia and Alzheimer’s treatment.	*In vivo* in rats treated with 100–300 mg/kg for 14 days.	[115]
*Abelmoschus esculentus* L. extract	Ethanol 85%.	Quercetin, isoquercetin, quercetin-3-*O*-rubinobioside, and myricetin with ROS reduction and neuronal cell proliferation properties.	*In vitro* with doses ranging between 10 and 75 µg/mL.	[116,117]
*Moringa oleifera* L. extract	Not reported.	Apigenin, kaempferol, quercetin, and isorhamnetin derivatives decreased lipid peroxidation in the brain.	*In vitro* up to 50 µg/mL and in vivo in rats at a dose of 20 mg/kg for 30 days.	[108]
	Not reported.	Quercetin, catechins, and epicatechins with neuroprotective effects.	Not reported.	[118]

**Table 3:** Plant flavonoids with anti-inflammatory potential.

Plant Species	Extraction Parameters	Anti-Inflammatory Flavonoid(s)	Assessment Procedure and Dose	Reference(s)
-	Not reported.	Naringenin, quercetin, myricetin, luteolin, kaempferol able to inhibit COX and nitric oxide synthase.	Not reported.	[118,119]
Aloe species	Ethanol, methanol, petroleum ether, chloroform, ethyl acetate, water.	Orientin and isovitexin modified the cytokine and C-reactive protein levels.	Not reported.	[84]
*Nelumbo nucifera* L. extract	80% methanol.	Lipoxygenase, COX-1, and COX-2 inhibition.	*In vitro*.	[101]
*Cymbopogon citratus* L. tea	Tea.	Anti-inflammatory.	*In vivo* in rats treated with 10% or 20% tea.	[121]
*Anchusa azurea* Mill. root extract	Ethanolic extract.	Catechin and quercetin-3-rhamnoside with anti-inflammatory properties.	*In vivo* in rats using doses of 200 or 400 mg/kg.	[123]
*Heliotropium strigosum* L. extract	Maceration in 70% ethanol for 24 h.	Quercetin with edema reduction properties.	*In vitro* in rats treated with 200–400 mg/kg root or leaf extracts.	[126]
*Heliotropium indicum* L. extract		Edema reduction properties.		
*Heliotropium crispum* L. extract		Quercetin, myricetin, and kaempferol with anti-inflammatory properties on brain cells.		

**Table 4:** Plant flavonoids against cardiovascular diseases.

Plant Species	Extraction Parameters	Cardio-Protective Flavonoid(s)	Assessment Procedure and Dose	Reference(s)
-	Not reported.	Naringenin inhibited ROS production and caspase-3 activity.	Not reported.	[120]
	Not reported.	Quercetin and derivatives with LDL lowering capacity.	Not reported.	[127]
	Not reported.	Kaempferol, catechins, and epicatechins with LDL lowering capacity.	Not reported.	[118]
*Lens culinaris* extracts	Ethanolic or aqueous extracts.	Quercetin and kaempferol with anticoagulant potential.	*In vivo* on rats treated with a dose of 10 µM.	[131]
	Not reported.	Catechin, gallocatechin, cyanidin, or delphinidin with hypocholesterolemic effect.	*In vivo* on rats treated with 100, 200 or 400 mg/kg.	[133]
*Ajuga iva* L. extract	Ethanolic or aqueous extracts.	Apigenin derivatives with anti-hypercholesterolemic effect.	Not reported.	[131]
	Aqueous extracts or ethanolic extracts (70%) produced at 60 °C after 12 h. Additional fractionation with n-hexane, dichloromethane, ethyl acetate, or butanol.	Myricetin, luteolin, quercetin, rhamnetin, apigenin derivatives, or naringin and naringenin, catechins, and epicatechins with anti-hypertensive effects.	*In vivo* on rats treated with 0.50–2 g/kg or 400 mg/kg for 14 days.	[134,135]
*Cymbopogon citratus* L. extract	Ethanolic or aqueous extracts.	Luteolin and apigenin with vasodilation properties	Not reported.	[131]
*Anchusa azurea* Mill.	Ethanol 60% for 6 h followed by ethanol 75% for 2 h at 90 °C for 2 h.	Rutin, quercetin, kaempferol, naringenin, and hesperidin beneficial upon chronic myocardial infarction.	*In vivo* on mice treated with 10, 30 or 50 mg/kg for 4 weeks.	[137]
*Heliotropium taltalense* L. extract	HPLC-grade methanol and ultrasonication for 1 h or aqueous extract obtained at 45 °C after 12 h.	Naringenin and quercetin reduced heart contractions; hypotensive effect associated with nitric oxide synthase.	Not reported.	[138]
*Trichosanthes kirilowii* L. extract	Ultrasound-assisted extraction at 40 kHz, for 1 h, at room temperature using ethanol and water (80:20).	Quercetin, kaempferol, luteolin, rutin, and apigenin derivatives exert vasodilation effects.	Not reported.	[139]
*Ephendrae herba* L. extract	Not reported.	Rutin, epicatechins, quercetin, hesperidin, and kaempferol have anti-hypertensive potential.	*In vivo* on rats treated with 0.10–10 mg/kg.	[122]
*Corchorus olitorius* L. extract	Not reported.	Isorhamnetin has hypotensive potential by decreasing ACE activity.	Not reported.	[142]

**Table 5:** Plant flavonoids with anti-diabetic potential.

Plant Species	Extraction Parameters	Anti-Diabetic Flavonoid(s)	Assessment Procedure and Dose	Reference(s)
-	Not reported.	Quercetin with anti-diabetic effect by modifying C-reactive protein and TNF-α levels.	Not reported.	[143]
*Cymbopogon citratus* L. extract	Methanolic extract.	Rutin possess anti-diabetic effect by downregulating pro-inflammatory cytokines.	*In vivo* in rats treated with doses of 100, 200, or 400 mg/kg.	[144,145]
*Heliotropium procubens* L. extract	Methanolic or aqueous extracts obtained after 24 h at room temperature.	Kaempferol, catechin derivatives, luteolin, and naringenin-7-*O*-glucosides have anti-diabetic properties via α-amylase and α-glucosidase inhibition.	*In vitro*.	[146]
*Equisetum arvense* L. extract	Maceration in ethanol 70% for 24 h at room temperature.	Kaempferol and quercetin glucosides or native luteolin and apigenin modified the metabolic pathways and exerted anti-diabetic properties.	*In vivo* in rats treated with 100 mg/kg for 6 weeks.	[147,148]
*Abelmoschus esculentus* L. beverage	Beverage.	Anti-diabetic effect by improved glucose tolerance.	*In vivo* in mice diet by supplementation with 0.52 mL/day.	[149]
*Corchorus olitorius* L. extract	Maceration at room temperature for 24 h using water, ethanol, or a mixture of water and ethanol in equal ratios.	Kaempferol, quercetin, luteolin, naringin, naringenin, quercetrin, and rutin have anti-diabetic properties due to antioxidant enzymes’ activity stimulation.	*In vitro* using concentrations between 10 and 500 μg/mL.	[150]
*Terminalia catappa* Linn. extract	Ethanolic (70%) extract obtained after 16 h at 70 °C.	Orientin and vitexin with anti-diabetic effects.	*In vivo* in *C. elegans* treated with doses between 6.25 and 25 µg/mL.	[152]
*Triticum* sp.	Not reported.	Cyanidin and peonidin glucosides or malvidin galactoside inhibited insulin-involving metabolic pathways.	*In vivo* in *C. elegans* treated with a dose of 100 µg/mL.	[153]

**Table 6:** Plant flavonoids with anticarcinogenic potential.

Plant Species	Extraction Parameters	Anticarcinogenic Flavonoid(s)	Assessment Procedure and Dose	Reference(s)
-	Not reported.	Epigallocatechin gallate has multiple anticarcinogenic properties. Quercetin reduced the proliferation of cervical and breast cancer cells. Luteolin has anticarcinogenic effects against colorectal and lung cancer. Apigenin was efficient in gastric, colorectal, or prostate cancer treatment.	*In vivo* in mice treated with 20 or 50 µg or 20 µg/mL, respectively.	[155]
Green tea (*Camellia sinensis*)	Tea.	Epigallocatechin gallate has inhibition potential on breast cancer cell proliferation.	*In vitro* test using doses of 5–50 µM/mL.	[156]
*Ulrica doica* L. and *Ulrica thunbergiana* L. extracts	Ethanolic or aqueous extracts and dichloromethane- or petroleum ether-based extracts.	Luteolin derivatives, quercetin, kaempferol, and isovitexin with anti-tumor, anti-proliferative, and apoptotic effects on malignant cells.	*In vitro*.	[100,118]
*Lens culinaris* Medik extracts	Acetonitrile and water extracts (70:30) obtained at room temperature after 19 h.	Kaempferol 3-*O*-arabinoside and apigenin glucoside were able to induce positive effects upon bone and muscle cancers.	*In vitro* tests using concentrations of 0.10 or 5 mg/mL.	[157]
	Not reported.	Kaempferol, quercetin, and myricetin inhibited breast cancer cells’ proliferation.	Not reported.	[158]
*Cymbopogon citratus* L. extract	Not reported.	Luteolin with antagonistic effect against prostate cancer cells.	*In vitro* test using doses between 30 and 1.000 µg/mL.	[121]
	Ethanolic extract.	Apoptotic effect on colon cancer cells.	*In vivo* in mice treated with 16 mg/kg.	[144,145]
*Equisetum arvense* L. extracts	Aqueous extract obtained at 100 °C after 6 min.	Luteolin, apigenin, kaempferol-3-*O*-glucoside, quercetin, and quercetin-3-*O*-glucoside had inhibitory effects against pancreatic and ovarian cancer.	*In vitro* tests involving 100 µg/mL to 1000 µg/mL.	[159,160]
-	Not reported.	Quercetin possesses inhibitory activity against malignant pancreatic cells.	*In vitro* and *in vivo*.	[161]
*Taxa fauna* L., *Taxa yunnanensis* L., *Taxa baccata* L., *Taxa brevifolia* L., *Taxa mairei* L. extracts	Not reported.	Apigenin, luteolin, kaempferol, myricetin, quercetin, and catechin derivatives with anticarcinogenic properties.	Not reported.	[162]
*Asplenium nidus* L. extract	Not reported.	Quercetin 7-*O*-rutinoside beneficial in hepatic cancer.	*In vitro*.	[161]

## Data Availability

No new data were created or analyzed in this study. Data sharing is not applicable to this article.
